# A guide to selecting high-performing antibodies for Stearoyl-CoA desaturase (SCD1) (UniProt ID: O00767) for use in western blot, immunoprecipitation, and immunofluorescence

**DOI:** 10.12688/f1000research.160217.1

**Published:** 2025-01-02

**Authors:** Vera Ruíz Moleón, Charles Alende, Maryam Fotouhi, Sara González Bolívar, Riham Ayoubi, Carl Laflamme, NeuroSGC/YCharOS/EDDU collaborative group, ABIF consortium

**Affiliations:** 1 Neurology and Neurosurgery, Montreal Neurological Institute-Hospital, Montreal, Québec, Canada

**Keywords:** O00767, SCD, SCD1, Steroyl-CoA desaturase, antibody characterization, antibody validation, western blot, immunoprecipitation, immunofluorescence

## Abstract

The enzyme stearoyl-CoA desaturase (SCD1) is a modulator of lipid metabolism by catalyzing the biosynthesis of mono-unsaturated fatty acids from saturated fatty acids. Understanding the specific mechanisms by which SCD1 plays in health and disease can provide novel insides in therapeutic targets, a process that would be facilitated by the availability of high-quality antibodies. Here we have characterized nine SCD1 commercial antibodies for western blot, immunoprecipitation, and immunofluorescence using a standardized experimental protocol based on comparing read-outs in knockout cell lines and isogenic parental controls. These studies are part of a larger, collaborative initiative seeking to address antibody reproducibility issues by characterizing commercially available antibodies for human proteins and publishing the results openly as a resource for the scientific community. While use of antibodies and protocols vary between laboratories, we encourage readers to use this report as a guide to select the most appropriate antibodies for their specific needs.

## Introduction

Stearoyl-CoA desaturase (SCD1) is a membrane-bound enzyme which catalyzes the rate-limiting step in the conversion of saturated fatty acids into mono-unsaturated fatty acids. ^1, 2^ The regulation of SCD1 is physiologically important as maintaining a proper ratio of saturated to monounsaturated fatty acids is essential for membrane fluidity. Disruption to this ratio can lead to pathological conditions, including cardiovascular disease, obesity, non-insulin dependent diabetes mellitus, hypertension, neurological diseases, immune disorders and cancer. ^2– 7^

SCD1 is of particular importance in Parkinson’s disease (PD) research as its inhibition has been found toto rescue α-Synuclein cytotoxicity and inclusion formation, both hallmarks of PD progression. The neurotoxic mechanisms underlying PD progression are not yet clearly defined. ^8– 10^ Mechanistic studies would be facilitated with the availability of high quality SCD1 antibodies.

This research is part of a broader collaborative initiative in which academics, funders and commercial antibody manufacturers are working together to address antibody reproducibility issues by characterizing commercial antibodies for human proteins using standardized protocols, and openly sharing the data. ^11– 13^ Here we evaluated the performance of nine commercial antibodies for SCD1 for use in western blot, immunoprecipitation, and immunofluorescence, enabling biochemical and cellular assessment of SCD1 properties and function. The platform for antibody characterization used to carry out this study was endorsed by a committee of industry academic representatives. It consists of identifying human cell lines with adequate target protein expression and the development/contribution of equivalent knockout (KO) cell lines, followed by antibody characterization procedures using most commercially available antibodies against the corresponding protein. The standardized consensus antibody characterization protocols are openly available on Protocol Exchange (DOI: 10.21203/rs.3.pex-2607/v1). ^14^

The authors do not engage in result analysis or offer explicit antibody recommendations. A limitation of this study is the use of universal protocols – any conclusions remain relevant within the confines of the experimental setup and cell line used in this study. Our primary aim is to deliver top-tier data to the scientific community, grounded in Open Science principles. This empowers experts to interpret the characterization data independently, enabling them to make informed choices regarding the most suitable antibodies for their specific experimental needs. Guidelines on how to interpret antibody characterization data found in this study are featured on the YCharOS gateway. ^15^

## Results and discussion

Our standard protocol involves comparing readouts from WT (wild type) and KO cells. ^16, 17^ The first step was to identify a cell line(s) that expresses sufficient levels of a given protein to generate a measurable signal using antibodies. To this end, we examined the DepMap transcriptomics database to identify all cell lines that express the target at levels greater than 2.5 log _2_ (transcripts per million “TPM” + 1), which we have found to be a suitable cut-off (Cancer Dependency Map Portal, RRID:SCR_017655). The HeLa expresses the SCD1 transcript at 6.7 log _2_ (TPM+1) RNA levels, which is above the average range of cancer cells analyzed. A *SCD* KO HeLa cells were obtained from Abcam ( Table 1).

**Table 1.** Summary of the cell lines used.

Institution	Catalog number	RRID (Cellosaurus)	Cell line	Genotype
Abcam	ab255448	CVCL_0030	HeLa	WT
Abcam	ab265220	CVCL_B2EP	HeLa	*SCD* KO

**Figure 1.**
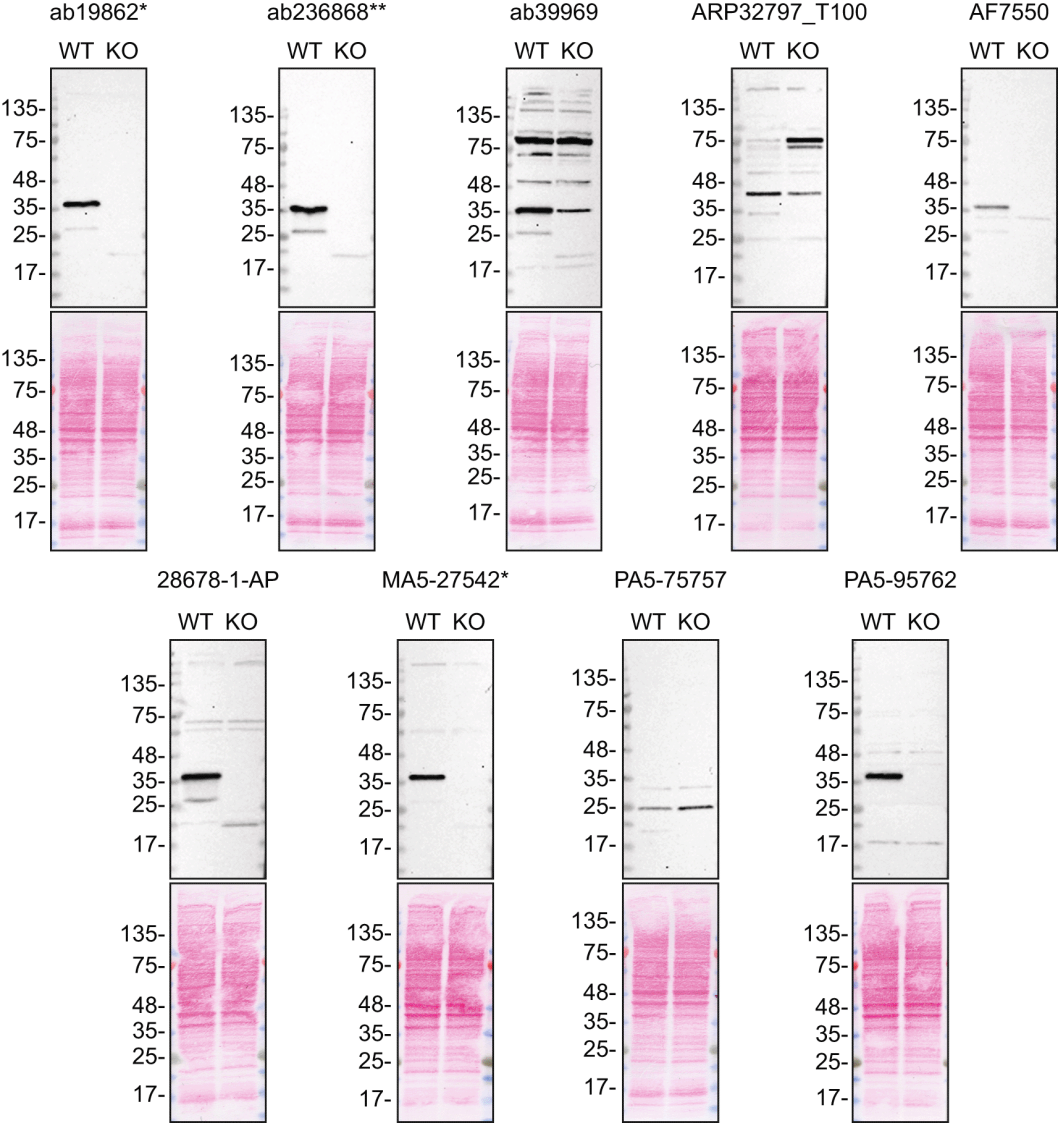
SCD1 antibody screening by western blot. Lysates of HeLa WT and *SCD* KO were prepared, and 35 μg of protein were processed for western blot with the indicated SCD1 antibodies. The Ponceau stained transfers of each blot are presented to show equal loading of WT and KO lysates and protein transfer efficiency from the acrylamide gels to the nitrocellulose membrane. Tris-Glycine 4-20% gels were used. Antibody dilutions were chosen according to the recommendations of the antibody supplier. An exception was given for antibody AF7550 which was titrated because the signal was too weak when following the supplier’s recommendations. Antibody dilution used: ab19862* at 1/1000, ab236868** at 1/1000, ab39969 at 1/1000, ARP32797_T100 at 1/1000, AF7550 at 1/200, 28678-1-AP at 1/1000, MA5-27542* at 1/1000, PA5-75757 at 1/200 and PA5-95762 at 1/1000. Predicted band size: 41.5 kDa *Monoclonal antibody, **Recombinant antibody.

For western blot experiments, WT and *SCD* KO protein lysates were ran on SDS-PAGE, transferred onto nitrocellulose membranes, and then probed with nine antibodies in parallel ( Table 2, Figure 1).

**Table 2.** Summary of the SCD1 antibodies tested.

Company	Catalog number	Lot number	RRID (Antibody Registry)	Clonality	Clone ID	Host	Concentration (μg/μl)	Vendors recommended applications
Abcam	ab19862 *	1057200-1	AB_445179	monoclonal	CD.E10	mouse	1.00	Wb, IP, IF
Abcam	ab236868 **	1007366-14	AB_2928123	recombinant mono	EPR21963	rabbit	0.61	Wb, IP, IF
Abcam	ab39969	1036585-6	AB_945374	polyclonal		rabbit	0.90	Wb
Aviva Systems Biology	ARP32797_T100	QC2226-43641	AB_841676	polyclonal		rabbit	1.00	Wb
Bio-Techne	AF7550	CGOP0121061	AB_3107036	polyclonal		sheep	0.20	Wb
Proteintech	28678-1-AP	00103543	AB_2923581	polyclonal		rabbit	0.40	Wb, IF
Thermo Fisher Scientific	MA5-27542 *	YH4004441A	AB_2723611	monoclonal	CD.E10	mouse	1.00	Wb, IP, IF
Thermo Fisher Scientific	PA5-75757	YJ4089139	AB_2719485	polyclonal		rabbit	1.00	Wb, IF
Thermo Fisher Scientific	PA5-95762	YJ4090059A	AB_2807564	polyclonal		rabbit	1.35	Wb, IF

Wb = western blot, IP = immunoprecipitation, IF = immunofluorescence.

* Monoclonal antibody.

** Recombinant antibody.

**Figure 2.**
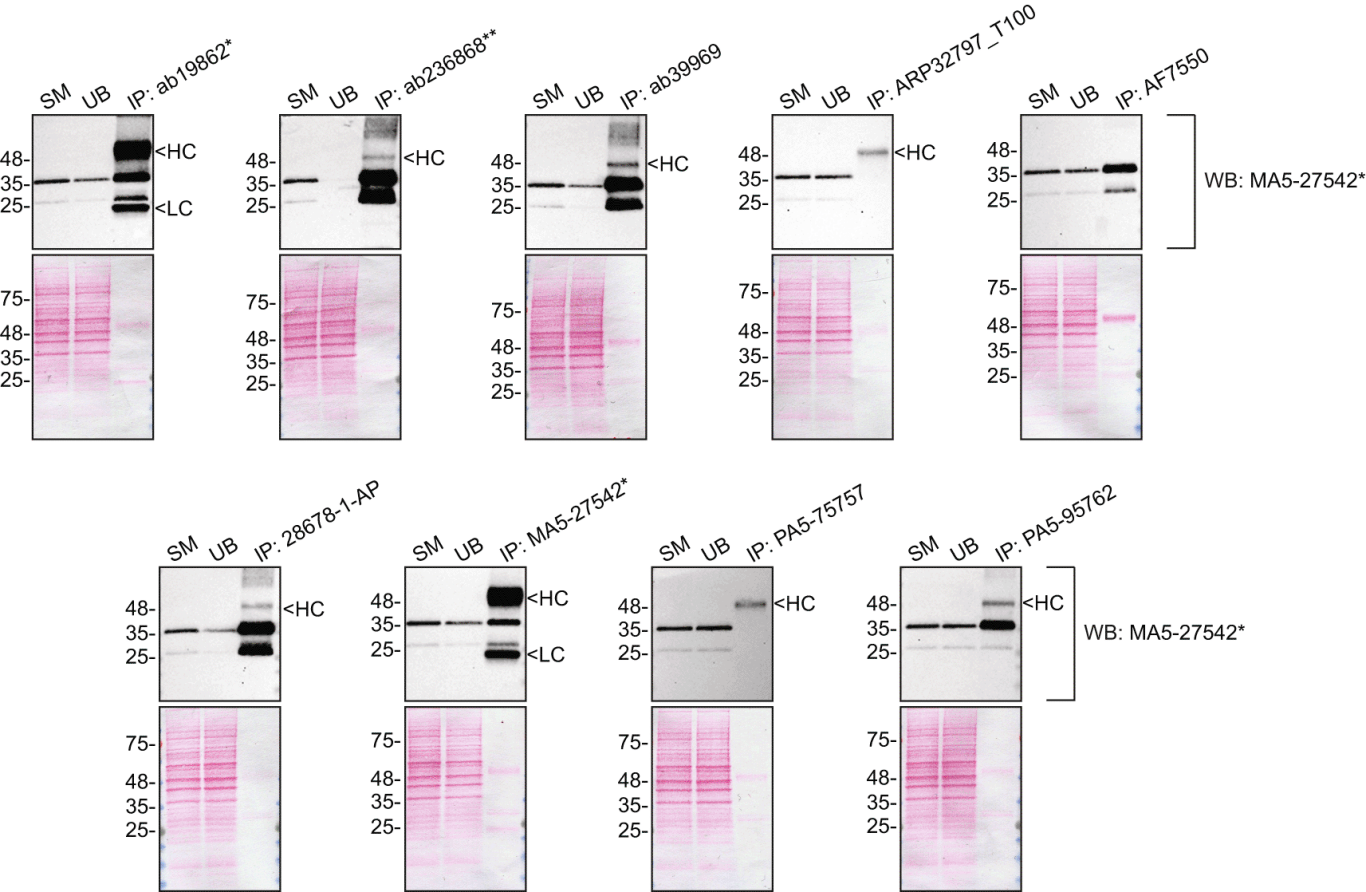
SCD1 antibody screening by immunoprecipitation. HeLa lysates were prepared, and immunoprecipitation was performed using 1 mg of lysate and 2.0 μg of the indicated SCD1 antibodies pre-coupled to Dynabeads protein A or protein G. Samples were washed and processed for western blot with the indicated SCD1 antibody. For western blot, MA5-27542* was used at 1/1000. Tris-Glycine 4-20% gels were used. The Ponceau stained transfers of each blot are shown. Predicted band size: 41.5 kDa. SM=4% starting material; UB=4% unbound fraction; IP=immunoprecipitate, HC= antibody heavy chain, LC= antibody light chain. *Monoclonal antibody, **Recombinant antibody.

We then assessed the capability of all nine antibodies to capture SCD1 from HeLa protein extracts using immunoprecipitation techniques, followed by western blot analysis. For the immunoblot step, a specific SCD1 antibody identified previously (refer to Figure 1) was selected. Equal amounts of the starting material (SM), the unbound fraction (UB), as well as the whole immunoprecipitate (IP) eluates were separated by SDS-PAGE ( Figure 2).

**Figure 3.**
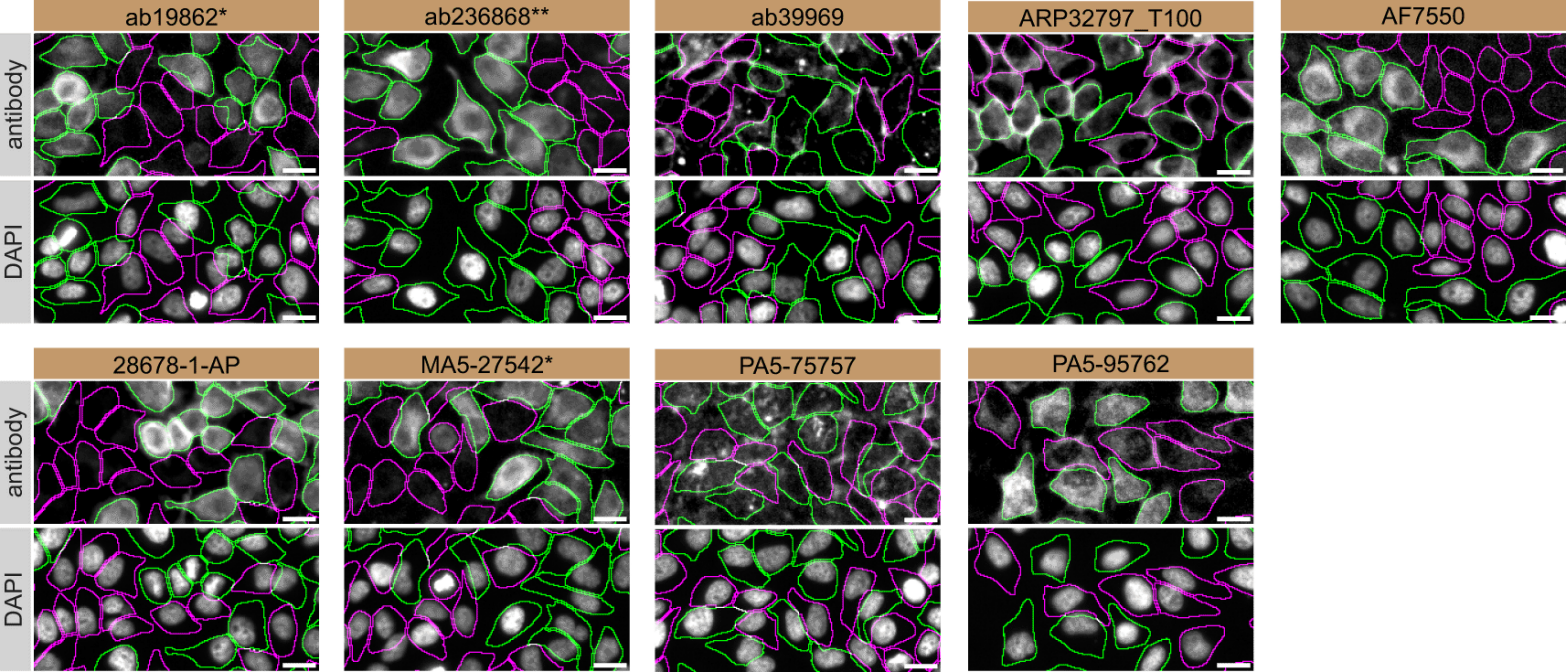
SCD1 antibody screening by immunofluorescence. HeLa WT and *SCD* KO cells were labelled with a green or a far-red fluorescent dye, respectively. WT and KO cells were mixed and plated to a 1:1 ratio on coverslips. Cells were stained with the indicated SCD1 antibodies and with the corresponding Alexa-fluor 555 coupled secondary antibody including DAPI. Acquisition of the blue (nucleus-DAPI), green (WT), red (antibody staining) and far-red (KO) channels was performed. Representative images of the merged blue and red (grayscale) channels are shown. WT and KO cells are outlined with green and magenta dashed line, respectively. When an antibody was recommended for immunofluorescence by the supplier, we tested it at the recommended dilution. The rest of the antibodies were tested at 1 and 2 μg/mL and the final concentration was selected based on the detection range of the microscope used and a quantitative analysis not shown here. Antibody dilution used: ab19862* at 1/1000, ab236868** at 1/600, ab39969 at 1/150, ARP32797_T100 at 1/500, AF7550 at 1/200, 28678-1-AP at 1/400, MA5-27542* at 1/1000, PA5-75757 at 1/1000 and PA5-95762 at 1/1300. Bars = 10 μm. *Monoclonal antibody, **Recombinant antibody.

For immunofluorescence, nine antibodies were screened using a mosaic strategy. First, HeLa WT and *SCD* KO cells were labelled with different fluorescent dyes in order to distinguish the two cell lines, and the SCD1 antibodies were evaluated. Both WT and KO lines imaged in the same field of view to reduce staining, imaging and image analysis bias ( Figure 3). Quantification of immunofluorescence intensity in hundreds of WT and KO cells was performed for each antibody tested, and the images presented in Figure 3 are representative of this analysis. ^14^

In conclusion, we have screened nine SCD1 commercial antibodies by western blot, immunoprecipitation, and immunofluorescence by comparing the signal produced by the antibodies in human HeLa WT and *SCD* KO cells. Several high-quality and renewable antibodies that successfully detect SCD1 were identified in all applications. Researchers who wish to study SCD1 in a different species are encouraged to select high-quality antibodies based on the results presented and investigate the predicted species reactivity of the manufacturer before extending their research.

## Method

The standardized protocols used to carry out this KO cell line-based antibody characterization platform was established and approved by a collaborative group of academics, industry researchers and antibody manufacturers. The detailed materials and step-by-step protocols used to characterize antibodies in western blot, immunoprecipitation and immunofluorescence are openly available on Protocol Exchange (DOI: 10.21203/rs.3.pex-2607/v1). ^14^

### Antibodies and cell line used

Cell lines used and primary antibodies tested in this study are listed in Tables 1 and 2, respectively. To ensure that the cell lines and antibodies are cited properly and can be easily identified, we have included their corresponding Research Resource Identifiers, or RRID. ^18, 19^

## Data Availability

Zenodo: Antibody Characterization Report for SCD1, https://doi.org/10.5281/zenodo.13891494. ^20^ Zenodo: Dataset for the SCD1 antibody screening study, https://doi.org/10.5281/zenodo.14502183. ^21^ Data are available under the terms of the Creative Commons Attribution 4.0 International license (CC-BY 4.0).

## References

[1] Enoch HG Catalá A Strittmatter P : Mechanism of rat liver microsomal stearyl-CoA desaturase. Studies of the substrate specificity, enzyme-substrate interactions, and the function of lipid. *J. Biol. Chem.* 1976;251(16):5095–5103. 10.1016/S0021-9258(17)33223-4 8453

[2] Ntambi JM : Regulation of stearoyl-CoA desaturase by polyunsaturated fatty acids and cholesterol. *J. Lipid Res.* 1999;40(9):1549–1558. 10.1016/S0022-2275(20)33401-5 10484602

[3] Kinsella JE Lokesh B Stone RA : Dietary n-3 polyunsaturated fatty acids and amelioration of cardiovascular disease: possible mechanisms. *Am. J. Clin. Nutr.* 1990;52(1):1–28. 10.1093/ajcn/52.1.1 2193500

[4] Jones BH Maher MA Banz WJ : Adipose tissue stearoyl-CoA desaturase mRNA is increased by obesity and decreased by polyunsaturated fatty acids. *Am. J. Phys.* 1996;271(1 Pt 1):E44–E49. 10.1152/ajpendo.1996.271.1.E44 8760080

[5] Li J Ding SF Habib NA : Partial characterization of a cDNA for human stearoyl-CoA desaturase and changes in its mRNA expression in some normal and malignant tissues. *Int. J. Cancer.* 1994;57(3):348–352. 10.1002/ijc.2910570310 7909540

[6] Habib NA Wood CB Apostolov K : Stearic acid and carcinogenesis. *Br. J. Cancer.* 1987;56(4):455–458. 10.1038/bjc.1987.223 3689663 PMC2001814

[7] Khoo DE Fermor B Miller J : Manipulation of body fat composition with sterculic acid can inhibit mammary carcinomas in vivo. *Br. J. Cancer.* 1991;63(1):97–101. 10.1038/bjc.1991.20 1989672 PMC1971644

[8] Fanning S Haque A Imberdis T : Lipidomic Analysis of α-Synuclein Neurotoxicity Identifies Stearoyl CoA Desaturase as a Target for Parkinson Treatment. *Mol. Cell.* 2019;73(5):1001–14.e8. 10.1016/j.molcel.2018.11.028 30527540 PMC6408259

[9] Bartels T Choi JG Selkoe DJ : α-Synuclein occurs physiologically as a helically folded tetramer that resists aggregation. *Nature.* 2011;477(7362):107–110. 10.1038/nature10324 21841800 PMC3166366

[10] Nicholatos JW Groot J Dhokai S : SCD Inhibition Protects from α-Synuclein-Induced Neurotoxicity But Is Toxic to Early Neuron Cultures. *eNeuro.* 2021;8(4):ENEURO.0166–ENEU21.2021. 10.1523/ENEURO.0166-21.2021 34301719 PMC8387157

[11] Ayoubi R Ryan J Biddle MS : Scaling of an antibody validation procedure enables quantification of antibody performance in major research applications. *elife.* 2023;12:12. 10.7554/eLife.91645 PMC10666931 37995198

[12] Carter AJ Kraemer O Zwick M : Target 2035: probing the human proteome. *Drug Discov. Today.* 2019;24(11):2111–2115. 10.1016/j.drudis.2019.06.020 31278990

[13] Licciardello MP Workman P : The era of high-quality chemical probes. *RSC Med. Chem.* 2022;13(12):1446–1459. 10.1039/D2MD00291D 36545432 PMC9749956

[14] Ayoubi R Ryan J Bolivar SG : A consensus platform for antibody characterization (Version 1). *Protocol Exchange.* 2024.

[15] Biddle MS Virk HS : YCharOS open antibody characterisation data: Lessons learned and progress made. *F1000Res.* 2023;12:1344. 10.12688/f1000research.141719.1 37854875 PMC10579855

[16] Laflamme C McKeever PM Kumar R : Implementation of an antibody characterization procedure and application to the major ALS/FTD disease gene C9ORF72. *elife.* 2019;8:8. 10.7554/eLife.48363 PMC6794092 31612854

[17] Alshafie W Fotouhi M Shlaifer I : Identification of highly specific antibodies for Serine/threonine-protein kinase TBK1 for use in immunoblot, immunoprecipitation and immunofluorescence. *F1000Res.* 2022;11:977. 10.12688/f1000research.124632.1 36415206 PMC9647147

[18] Bandrowski A Pairish M Eckmann P : The Antibody Registry: ten years of registering antibodies. *Nucleic Acids Res.* 2023;51(D1):D358–D367. 10.1093/nar/gkac927 36370112 PMC9825422

[19] Bairoch A : The Cellosaurus, a Cell-Line Knowledge Resource. *J. Biomol. Tech.* 2018;29(2):25–38. 10.7171/jbt.18-2902-002 29805321 PMC5945021

[20] Ruiz Moleon V Alende C Fotouhi M : A guide to selecting high-performing antibodies for Stearoyl-CoA desaturase (SCD1) (UniProt ID: O00767). *Zenodo.* 2024. 10.5281/zenodo.13891494 PMC12246776 40657179

[21] Laflamme C : Dataset for the SCD1 antibody screening study.[Dataset]. *Zenodo.* 2024. 10.5281/zenodo.14502183

