# Supplementary material for: A guide to selecting high-performing antibodies for Stearoyl-CoA desaturase (SCD1) (UniProt ID: O00767) for use in western blot, immunoprecipitation, and immunofluorescence
Source: F1000Res. 2025 Jun 13;14:10. Originally published 2025 Jan 2. [Version 2] doi: 10.12688/f1000research.160217.2 (PMC12246776; doi:10.12688/f1000research.160217.2)
Supplement: Peer review history

# Reviewer report: Reviewer response for version 2

**DOI:** 10.5256/f1000research.183557.r392564

**Published:** 2025-07-10

**Authors:** Deborah Moshinsky

**Affiliations:** 1 Institute for Protein Innovation, Boston, Massachusetts, USA

The authors tested 9 commercially available antibodies to SCD1 (stearoyl-CoA desaturase) for activity in Western Blot, Immunoprecipitation (IP) and immunofluorescence (IF).  They first found a cell line expressing the protein and generated a knockout version.  Then they utilized standardized, openly available protocols for Western, IP and IF.  The results were clear and the methodology easy to follow.  Table 3 is useful to assist in the interpretation of the data.  However, for the Western Blot examples in Table 3, the situation where multiple bands including the target are present in the WT and multiple bands but lacking the target is present in the KO is labeled as a 'successful antibody.'  I recommend that this be clarified, as one could argue that an antibody that detects it's target along with other proteins is non-specific and therefore not a 'successful' antibody.  Perhaps successful but non-selective or another qualifying term would be more suitable.

Are sufficient details of methods and materials provided to allow replication by others?

Yes

Is the rationale for creating the dataset(s) clearly described?

Yes

Are the datasets clearly presented in a useable and accessible format?

Yes

Are the protocols appropriate and is the work technically sound?

Yes

Reviewer Expertise:

Antibody characterization and validation

I confirm that I have read this submission and believe that I have an appropriate level of expertise to confirm that it is of an acceptable scientific standard.

# Reviewer report: Reviewer response for version 1

**DOI:** 10.5256/f1000research.176074.r358436

**Published:** 2025-02-05

**Authors:** Michael L Garelja

**Affiliations:** 1 University of Otago, Dunedin, New Zealand

This manuscript, aiming to investigate the utility of a range of SCD1 antibodies is extremely important, and I applaud the initiative. Identification of antibodies that can correctly detect their target is of incredible importance to the entire field and highlighting that not all antibodies work as advertised is critical to ensuring that researchers can perform robust science.

 Points that need be addressed for this work to be indexed.

- Are there RRID’s associated with these antibodies? This applies for both the primary antibodies, and the secondary antibodies. Secondary antibodies also require lot numbers.
- As antibody concentrations can change between batches, it would be more informative to list the concentrations used in the experiments rather than the dilutions.
- A summary table to show how the different antibodies work under different experimental techniques would be extremely useful. I understand that the resource cannot make recommendations, but having a summary of how each antibody would help others interpret results.
- Are there any known splice variants of this target? There is a consistent band of approximately 25 kDa, that also seems to be lower when knocked out. Any insight as to why the detected band differed from the estimated molecular weight would also be useful. Has this particular ladder been compared to other ladders to ensure that it runs as expected under these experimental conditions?

Further information for discussion.

- The researchers have used a cell-line which highly expresses the target. Is it possible to work with cells that have lower expression levels? If not, these needs to be addressed as a limitation. Testing the panel of “useful” antibodies against more cell lines with variable SCD1 expression to show a limit of detection would be of high utility.
- Listing the epitopes that the antibodies were raised against would be of high value. One of the pillars of antibody validation is to use two antibodies that have been raised against different epitopes, so having that information readily available would help researchers in their mission to perform good science.
- The paper should acknowledge that there may be methodological optimizations that could improve antibody function.
- Figure 3 – is it possible to show to green and red stained cells in a separate panel? The outlines are useful, but showing that the cells have similar morphologies (or not!) would be interesting in the context of SCD1 biology.
- Why were these antibodies chosen, as opposed to the wider range of antibodies available? This is not a criticism, as the work is valuable regardless, but some information as to how these antibodies were selected would be interesting.

Are sufficient details of methods and materials provided to allow replication by others?

Partly

Is the rationale for creating the dataset(s) clearly described?

Yes

Are the datasets clearly presented in a useable and accessible format?

Yes

Are the protocols appropriate and is the work technically sound?

Yes

Reviewer Expertise:

CGRP, migraine, pharmacology

I confirm that I have read this submission and believe that I have an appropriate level of expertise to confirm that it is of an acceptable scientific standard, however I have significant reservations, as outlined above.

# Reviewer report: Reviewer response for version 1

**DOI:** 10.5256/f1000research.176074.r355980

**Published:** 2025-01-17

**Authors:** Cecilia Williams, Matilda Holm

**Affiliations:** 1 KTH Royal Institute of Technology,, Stockholm, Sweden; 2 Protein Science, KTH Royal Institute of Technology (Ringgold ID: 7655), Stockholm, Stockholm County, Sweden

The data note by Ruiz Moleon et al. provides a high-quality antibody validation guide for the protein SCD1. The guide is part of the YCharOS initiative that uses isogenic knockout cell lines and multiple antibody-dependent assays to investigate antibody specificity and selectivity in a structured manner. It is an important effort that can improve the reproducibility of biomedical research, and this guide is highly useful for researchers focusing on SCD1. The authors show that 3-4 out of 9 antibodies do not work in most or any of the assays, whereas the rest are more or less well-performing, using the cells and conditions applied in the protocol. This is critical information that will be of high value to the research community. For enhanced usability, clarification, and stringency of the guide and to facilitate the interpretation and reproducibility, I have the below recommendations.

**Major comments**

- The introduction would benefit from including basic information regarding the protein, as this would facilitate the interpretation of the data. Such as the known or predicted subcellular localization of SCD1, its calculated molecular weight, and if any splice variants are known.
- The rationale, as stated (p.3, 2 ^nd^ paragraph: “ *Mechanistic studies would be facilitated with the availability of high-quality SCD1 antibodies*”) should be better supported. Is there a documented lack of high-quality SCD1 antibodies? Or, is this an assumption based on the general problem with the reproducibility of antibody-based research?
- The sentence “ *using most commercially available antibodies against the corresponding protein” (p. 3, 3 ^rd^paragraph)* is likely not correct. Perhaps the authors intended to state “ *renewable* antibodies” as the initiative states in other publications. In this guide, specifically, the authors include 9 commercial (renewable and non-renewable) SCD1 antibodies, but there are as many as 274 antibodies from 32 providers directed towards human SCD1 listed in Antibodypedia.
- A statement of whether the SCD1 gene in the cells (HeLa WT) has indeed been confirmed to be wild type (i.e., not mutated) should be added. It would also be useful to include which (if any) splice variant transcripts are expressed in these cells.
- Table 1: The lot (or batch) numbers for the antibodies must be listed. This is essential information and is especially important in an antibody validation guide.
- It should be stated whether replicate experiments were performed.
- It is unclear how the antibody used for the western blot of the IPs was chosen (p. 3, 2 ^nd^ last paragraph: “ *a specific antibody was chosen*”). Were both specificity and selectivity considered? Were the species the antibodies were generated in used as criteria (to avoid detection of heavy and light chains in the next step)? Or was the selection random out of the sufficiently specific antibodies? If so, how many and which antibodies were deemed to be sufficiently specific?
- Fig 2: Considering that antibodies from different species appear specific in WB, it seems like a missed opportunity to not take species into account to generate clearer western blot data of the IPs (i.e., avoiding additional bands of heavy and light antibody chains, such as when using mouse-generated antibodies in both IP and WB and detecting them with an anti-mouse secondary antibody).
- Fig. 1 and 2: It should be specified which secondary antibody was used in the western blots.
- Fig. 3: The legend statement that “ *Representative images of the merged blue and red (grayscale) channels are shown.*” appears incorrect. The blue (DAPI) is shown separately from the red (antibody staining); they are not merged in the figure. It would also be clearer if the images showed the actual colors (blue for DAPI and red for SCD1 antibody) instead of grey for both.
- Only one cell line is used, which is a limitation of the initiative. Considering that HeLa cells express unusually high SCD1 levels (as stated in the text), the antibodies may work less well at lower SCD1 levels. They could also bind to other proteins expressed in other cells but not HeLa. It would be useful if the antibodies (the ones deemed to be of high quality) were additionally analyzed by western blot on a panel of cell lines with varying mRNA expression of *SCD1*. This would indicate their performance at different levels of the target protein and in more than one cell line.
- Although the authors state that they do not engage in result analysis, they do (to select a specific antibody for WB of the IP, and they conclude that several antibodies successfully detect SCD1), but they refrain from clearly stating which these antibodies are. A table, or a statement, summarizing which antibodies bind intended and unintended targets in the different assays (under the specific conditions used) would allow greater usability, including for researchers starting their work (such as postdocs and PhD students). This would also facilitate an overview of the application-dependent performance (again, under the specific conditions used).

**Minor comments:**

- The assay immunofluorescence (IF) is typically used for tissue analysis. For analysis of cells and cell lines, as is the case here, the term immunocytochemistry is better suited.
- UniProt ID may not need to be stated in the title (could be moved to abstract or introduction)
- p.3, 3 ^rd^ paragraph: It should be clarified if the target protein expression relates specifically to *endogenous *expression or whether cell lines with recombinant expression were included.
- A statement about mycoplasma testing and cell line authentication should be included.
- It would be beneficial to include which epitope each antibody is directed toward (or raised against).
- Fig. 1 and 2: The exposure time(s) applied for the western blots could be included.
- Fig 2: Although the figure legend clearly states which antibody was used for WB of all IPs, the figure itself gives the impression that it may have been used only for the two indicated IPs. It might be better to remove this from the image or place it differently.
- p. 5, first paragraph: “ *Quantification of ... in hundreds of WT and KO cells*” is vague. The number (or range) of actually quantified cells should be stated.
- Fig. 3: The figure legend should specify which magnification was used for the IF images.
- It would be beneficial to highlight that the antibodies ab19862 and MA-27542 are from the same clone and should be the same. It would be pedagogical to place them side by side.
- Page 5, final paragraph: “ *Several high-quality and renewable antibodies that successfully detect SCD1 were identified in all applications”. *As only 3 of the tested antibodies were renewable (monoclonal or recombinant), and two of them were from the same clone (= same antibody), the word “several” seems a stretch. The word “renewable” should probably be removed.
- Several spelling/grammar errors (p.3 a “to” too much p.3, p. 3 “were run “ instead of “were ran”, wrongly placed commas, etc.)
- The published method protocol Ayoubi et al., Nature Protocol (2024) should likely be referenced.
- Author roles: It is not stated who drafted the manuscript, nor if all authors commented on it.

Are sufficient details of methods and materials provided to allow replication by others?

Partly

Is the rationale for creating the dataset(s) clearly described?

Yes

Are the datasets clearly presented in a useable and accessible format?

Partly

Are the protocols appropriate and is the work technically sound?

Yes

Reviewer Expertise:

nuclear receptors, transcriptomes, antibody validation, cancer

We confirm that we have read this submission and believe that we have an appropriate level of expertise to confirm that it is of an acceptable scientific standard, however we have significant reservations, as outlined above.
